# Timely mental health services contribute to the containment of COVID-19 pandemic in China

**DOI:** 10.1186/s41256-020-00168-x

**Published:** 2020-09-03

**Authors:** Ning Zhang, Kankan Wu, Weidan Wang

**Affiliations:** 1 School of Public Health and the Second Affiliated Hospital of Zhejiang University School of Medicine, Hangzhou, China; 2 Institute of Psychology, Chinese Academy of Sciences, Beijing, China; 3 Tongde Hospital of Zhejiang Province, Hangzhou, China

## Abstract

The COVID-19 pandemic is the most severe public health crisis in the 21st century. The pandemic not only posed great challenges to people's physical health but also induced wide-ranging impacts on mental health of infected and suspected patients, frontline healthcare workers, and the general public whose normal life was disrupted by the pandemic. In this commentary, we outline the initiatives and coordinated efforts on providing timely mental health services after the pandemic outbreak in China, including understanding the mental health impact of COVID-19, prioritizing and coordinating mental health services along with medical services in the efforts to contain the pandemic, initiating and implementing specific measures to improve mental wellbeing of frontline healthcare workers, and increasing the accessibility of mental health services to the general public. Theses services, along with other coordinated efforts, contribute to the containment of COVID-19 pandemic in China and could be valuable for other countries to take proactive measures to mitigate the mental health impacts of the pandemic now and in the future.

## Background

The coronavirus disease 2019 (COVID-19) pandemic is the most severe public health threat to people around the world in the twenty-first century. According to the information released by Johns Hopkins University, by 6:30 am, July 31st, 2020, 17,153,442 patients had been infected by COVID-19, of whom 669,701 died due to the infection [1]. Since the outbreak of COVID-19, both the central and local governments of China initiated and implemented strict restrictions and policies on limiting unnecessary public gatherings, contact tracing, epidemiological investigation, and advocating people to engage in health protective behaviors (e.g., wearing face masks while going out, washing hands more frequently, practicing social distancing, etc.) to reduce human-to-human transmission of COVID-19. These strategies contributed greatly to the containment of the COVID-19 pandemic in China [2].

As other infectious diseases outbreak such as SARS, MERS and Ebola, the COVID-19 pandemic induced wide-ranging impacts on mental health of confirmed and suspected patients, frontline healthcare workers, and the general public, such as fear, anxiety, depression, insomnia, and suicidal ideality [3, 4]. As reported by the MIT Technology Review, the COVID-19 pandemic is the first social-media “infodemic”, which amplified mental health stress among those being directly impacted by the pandemic and the general public with high exposure to information and misinformation regarding the pandemic [5]. However, what’s different from previous infectious disease pandemic happened in China is that timely mental health services, including psychological hotlines, mental health screening and counselling, psychotherapy, and psychiatric services, were initiated and implemented shortly after the outbreak of COVID-19. In this commentary, we outline the initiatives and coordinated efforts on providing timely and continuous mental health services since the outbreak of COVID-19. These timely mental health services empower the whole society to work collaboratively to contain the COVID-19 pandemic in China.

## Understanding the mental health impact of COVID-19

Shortly after the outbreak of COVID-19, researchers in mental health, psychiatry, counseling and clinical psychology, public health, social psychology and social work are conducting emergent research on understanding and monitoring the psychological impact of the COVID-19 pandemic on confirmed and suspected patients, frontline healthcare workers, those experienced quarantine, and the general public. By using social media to monitor social emotions during the epidemic, researchers from the Institute of Psychology of the Chinese Academy of Sciences are working on developing effective strategies to provide tailored mental health services to those in need [6]. As revealed by a national longitudinal survey on social emotions during the epidemic by the Institute of Sociology, of the Chinese Academy of Social Sciences, experience of negative emotions such as worry, fear, anger, and panic were decreasing while experience of calm and hope were increasing, suggesting that social emotions during the COVID-19 pandemic already bounced back to normal [7]. Of course, there are still areas to improve to coordinate the available resources more efficiently and to provide timely mental health services during this public health emergency. Leading experts on psychological health services advocated on building and improving the national social psychological service system to be prepared for such public health emergencies in the future.

## Prioritizing the provision of mental health services to targeted populations

Shortly after the outbreak of COVID-19, the central government prioritized timely mental health services for those being directly and indirectly impacted by the pandemic. The National Health Commission of China released guidelines on providing emergent mental health assistances and psychological crisis interventions during the pandemic [8]. Specifically, the guidelines paid special attention to providing timely psychological screening and tailored mental health services to four target groups of people: (1) infected patients and frontline healthcare workers; (2) suspected patients and close contacts of infected patients who had to stay in quarantine; (3) close contacts of the first two categories, and other personnel getting involved in the efforts to contain the pandemic; (4) other vulnerable groups (e.g., elders, people with mental health disorders, left-behind children, etc.) and the general public.

In response to the advocate on providing timely mental health services during the pandemic, professional organizations such as the Chinese Psychological Society and its sub-working committees initiated online programs shortly after the pandemic to train professionals and volunteers to provide psychological assistances and interventions (e.g., establishing hotlines for psychological assistance, and providing online psychological screening, feedback, and support) to people in need. On March 2nd, 2020, president Xi reemphasized the importance of providing timely and continuous psychological counseling, psychotherapy, and other needed mental health services to confirmed and suspected patients, their families, and the general public. This is the first time that the National Health Commission released specific guidelines on mental health services after a public health emergency in China. These guidelines contributed to the coordination of mental health services and resources around the country.

## Improving the mental wellbeing of healthcare workers

**Fig. 1:**
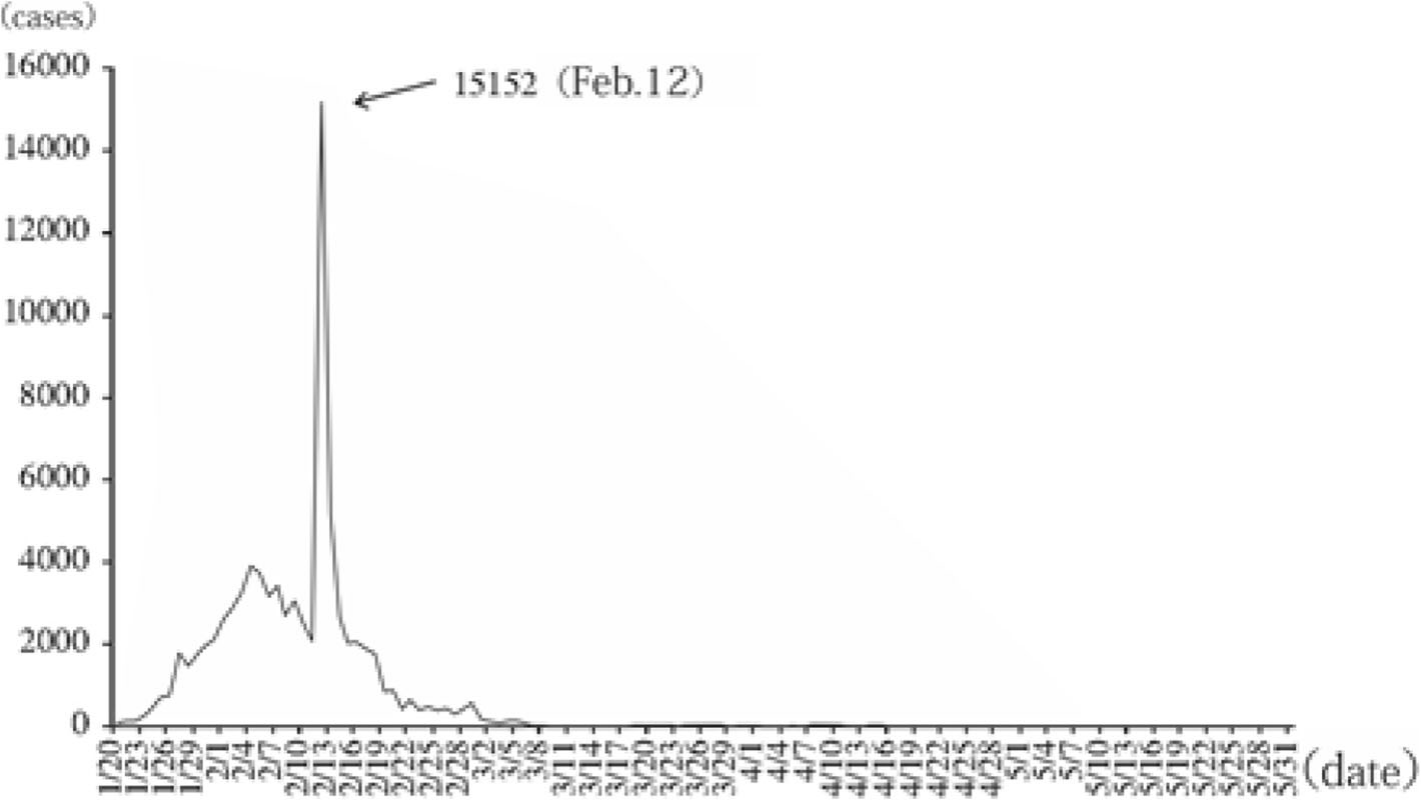
Daily Confirmed New Cases within Mainland China. Data source: Fighting COVID-19: China in Action, Available from http://www.xinhuanet.com/english/2020-06/07/c_139120424.htm.

Special efforts were initiated and implemented on protecting the mental health of healthcare professionals and their families. Healthcare workers are the backbone in the battle against the COVID-19 pandemic, in response to this public health emergency, more than 42,000 healthcare professionals around the country were sent to help Wuhan and other major cities in Hubei to contain the COVID-19 pandemic in China. With increasing numbers of patients being infected and diagnosed with COVID-19 during the peak period of the pandemic (from late January to late February, see Fig. 1), healthcare professionals experienced a high level of stress due to limited working conditions and medical resources, long working shifts and high risk of infection. The National Health Commission of China released specific guidelines on improving the working conditions and mental wellbeing of healthcare professionals and their families during the COVID-19 pandemic [9].

Initiatives on providing timely mental health services for healthcare workers included sending mental health teams composed of psychiatrists, mental health nurses, and clinical psychologists to work onsite with healthcare workers, providing 24/7 hotlines and web-based mental health services, launching apps for mental health screening, setting up independent resting spaces for healthcare workers, providing support to families of healthcare workers, maintaining and rebuilding social support networks for healthcare workers. These timely meatal health services reduced stress and enhanced resiliency among healthcare workers, which in turn, improved the efficiency of the collective efforts in containing the COVID-19 pandemic in China [4].

## Making mental health services accessible to the general public

Due to the emergent policies on social distancing to reduce human-to-human transmission of COVID-19, it is not feasible for people to seek help for mental health in person. Therefore, it is important to make mental health services accessible to those in need. Since the outbreak of COVID-19 pandemic, scientists from clinical psychology, health psychology, social psychology, social work, psychiatry and other relevant disciplines from universities, research institutes, mental health centers within hospitals around the country have worked collaboratively in preparing mental health education materials, giving online lectures on mental health adjustment and resiliency, and sharing mental health education materials through social media (e.g., WeChat) to the general public.

Mental health centers affiliated with leading research universities such as the Six Hospital of Peking University published books and guidelines on assisting the general public to monitor their mental health status and take proactive measures to improve their mental health and enhance resiliency. More than 600 hotlines for mental health services were established since the outbreak. Thousands of online mental health support groups were established to improve people’s mental health with support from registered counsellors. Professional organizations such as the Chinese Psychological Society also leveraged the advanced internet infrastructure and online healthcare platforms to provide online mental health consultation and to increase the accessibility of mental health resources [10].

## Conclusion

During the COVID-19 pandemic, governments, professional organizations, research institutes and universities, psychiatry departments and mental health centers across China have swiftly initiated guidelines, mental health services, and campaigns to address mental health needs for healthcare workers, infected/suspected patients and their families, those experiencing quarantine, and the general public [8, 9]. Timely mental health services are included as an essential component of the public health emergency system and are contributing to improving the efficiency of containing the COVID-19 pandemic in China. Due to the spreading of COVID-19 around the world and it’s profound impact on the mental health of healthcare workers, infected and suspected patients, and the general public, efforts on addressing mental health needs and building resiliency should be strengthened both during and after the COVID-19 pandemic to relieve the acute and long term impacts of the pandemic around the world.

## Data Availability

Not applicable.

## Abbreviations

COVID-19: Coronavirus disease 2019
MERS: Middle East Respiratory Syndrome
SARS: Severe Acute Respiratory Syndrome
